# Editorial: Investigating the Mechanism of TMS Using Brain Imaging Methods

**DOI:** 10.3389/fnins.2022.936219

**Published:** 2022-05-20

**Authors:** Bin Zhang, Xin Luo, Yuping Ning, Jijun Wang, Yu-Feng Zang

**Affiliations:** ^1^Affiliated Brain Hospital of Guangzhou Medical University, Guangzhou, China; ^2^Shanghai Mental Health Center, Shanghai Jiao Tong University School of Medicine, Shanghai, China; ^3^Center for Cognition and Brain Disorders, Institutes of Psychological Sciences, Hangzhou Normal University, Hangzhou, China

**Keywords:** repetitive transcranial magnetic stimulation (TMS), brain imaging, MRI, EEG, mechanism

Neuromodulation is an emerging therapeutic approach, in which transcranial magnetic stimulation (TMS) are commonly used in clinical practice (Dayan et al., 2013; Flöel, 2014; Voigt et al., 2021). To develop a better understanding of the neurophysiological basis of those treatments, brain imaging has been carried out (Eldaief et al., 2011). Speaking of neuroimaging, great signs of progress have been made in this realm and its techniques, leading to the improvement of the time and space resolution and overall accuracy of imaging. The growth of the human neuroimaging literature has led to major advances in understanding the mechanism of the neuromodulation technique. This Research Topic provides some new insights into the mechanism of neuromodulation techniques based on brain imaging methods. Broadly, this includes studies focusing on TMS, transcranial electrical stimulation (tES), magnetic resonance imaging (MRI), and electroencephalogram (EEG).

Recent years have witnessed the rising popularity of TMS, an innovative non-invasive form of brain stimulation, which works by creating a focal magnetic field that changes brain activity in both transient and prolonged ways (Hallett, 2000, 2007). Being safe, well-tolerated and effective, the new technique is increasingly attracting the attention of researchers and clinicians and is used in several neuroscience research domains such as cognition, memory, and affective processing (Pitcher et al., 2021). There is currently a limited understanding of the neurophysiological basis of TMS despite the prevalence of TMS in neuroscience research. A real-time evaluation of neuronal activity induced by TMS will contribute to a deeper understanding of its mechanism.

Due to the spatiotemporal resolution of functional MRI, neurofeedback with real-time functional MRI is particularly suited to studying the mechanism of TMS. Coupled with TMS, however, functional magnetic resonance imaging (fMRI) always produces artifacts. Based on a transmit-receive (Tx/Rx) single-channel birdcage head rf coil and a 20-channel head rf coil in 3T-MRI scanner, Caparelli et al. accessed this multimodal tool for improving the quality of real time-fMRI images. They have shown that artifact reduction can be achieved by using a large single-channel radio frequency (rf) coil with an axial imaging orientation and a 100 ms safety interval between TMS pulses and imaging acquisition.

In addition to being used to study mechanisms, MRI is crucial in improving the efficacy of TMS (Cash et al., 2020, 2021). Sophisticated neuroimaging technology facilitates the accuracy of localizing the TMS stimulation targets. High-resolution structural magnetic resonance imaging can provide quantitative measurements of morphological and geometric features, which is critically important for navigating brain stimulation (Lu 2021). The efficacy of TMS highly depends on the stimulus target (Fox et al., 2012; Jing et al., 2020; Fitzgerald, 2021). Zhang et al. reviewed different TMS location methods for depression. Of note, MRI-guide method mentioned in the study can be used for identifying individual TMS targets. Based on methylazoxymethanol acetate (MAM) rats, Guo et al. found that the visual cortex (VC) might be a novel TMS target for adolescent schizophrenia. Collectively, these studies outline a critical role for MRI in the stimulus targets.

As a non-invasive stimulation, TMS can be conducted to probe neurophysiological processes in the brain. EEG can directly measure neuronal responses. The combination of TMS and EEG, as a non-invasive measure for brain activity, has proven to be a promising method. However, a limitation of the technology is the redundant information recorded by EEG. Therefore, founding the valuable components is an important aspect of TMS-EEG.

TMS-EEG applied to the primary motor cortex provides TMS-evoked potentials (TEPs) for cortical excitability. The second prominent negative TEP peak is N100. N100 is characterized by a negative peak at a latency of ~100 ms after the TMS pulse, which is a commonly studied EEG component (Bender et al., 2005). Roos et al. report data from 12 healthy participants which shows the N100 signal triggered by TMS in the primary motor cortex (M1) might result from both the anatomical properties of M1 and the local excitability of the surrounding cortical areas. Therefore, the detection of the local cortical excitability differences may be suitable for N100 amplitudes. Besides, Jarczok et al. suggest focusing on the lateralized TEP component of EEG, which is the late negative deflections corresponding to the N100 in motor cortex stimulation, will contribute to a deeper understanding of TEP.

TES, another commonly used neuromodulation technique, increases motor learning through stimulation of the M1 area (Gao, Cavuoto, Schwaitzberg et al.). However, most studies have only focused on simple unimanual motor sequence learning. Gao, Cavuoto, Dutta et al. recruited 14 medical students with no operational experience and randomly divided them into a tDCs group and a sham group, showing that tDCs significantly increased motor learning ability and reduced performance errors in complex surgical motor skill learning. Employing the fNIRS to acquire brain activation, they found that contralateral M1 and PFC brain activation were decreased and SMA was increased by tDCS. This may be the mechanism for the increased ability of motor learning.

Taken together, these studies demonstrate a powerful combination of neuroimaging and neuromodulation. The use of the multimodal tool is an important issue for studying and understanding neuromodulation. Further work is required to focus on the application of high spatial-temporal resolution multimodal tools to explore neurobiological mechanisms and improve the efficacy of neuromodulation.

## Author Contributions

All authors listed have made a substantial, direct, and intellectual contribution to the work and approved it for publication.

## Conflict of Interest

The authors declare that the research was conducted in the absence of any commercial or financial relationships that could be construed as a potential conflict of interest.

## Publisher's Note

All claims expressed in this article are solely those of the authors and do not necessarily represent those of their affiliated organizations, or those of the publisher, the editors and the reviewers. Any product that may be evaluated in this article, or claim that may be made by its manufacturer, is not guaranteed or endorsed by the publisher.
